# Multiple Organ Dysfunction Secondary to Herpes Simplex Virus -1 Reactivation After Treatment With Dexamethasone and Sarilumab for Covid-19 Disease

**DOI:** 10.2478/jccm-2023-0010

**Published:** 2023-05-08

**Authors:** Thomas Roe, Sam Waddy, Nikitas Nikitas

**Affiliations:** University Hospitals Plymouth NHS Trust, Plymouth, Devon, United Kingdom

**Keywords:** coronavirus, herpes simplex virus, reactivation, intensive care, multiple organ dysfunction

## Abstract

**Introduction:**

The immunological response to the SARS-CoV-2 virus and the treatment of COVID-19 disease present a potential susceptibility to viral reactivation, particularly Herpes simplex virus-1 (HSV-1).

**Case Presentation:**

A 49-year-old female presented to hospital with severe COVID-19 pneumonitis and was given sarilumab and dexamethasone. She was intubated and ventilated in the intensive care unit (ICU) and initially demonstrated biochemical and clinical evidence of improvement. This was followed by a severe acute deterioration in respiratory, renal, and cardiovascular function, accompanied by a vesicular rash on the face. Polymerase chain reaction confirmed HSV-1 reactivation and treatment with acyclovir was commenced. After 49 days in ICU the patient was successfully weaned from all organ support, and she made a satisfactory recovery.

**Conclusions:**

HSV-1 reactivation is common in COVID-19 and likely contributes to poorer clinical outcomes. The mechanism causing susceptibility to viral reactivation is not clearly defined, however, the development of critical illness induced immunosuppression via dysfunction of interferon and interleukin pathways is a likely mechanism. This effect could be perpetuated with immunosuppressant medications, although further research is needed to characterise this phenomenon.

## INTRODUCTION

Severe acute respiratory syndrome coronavirus 2 (SARS-Cov-2) giving rise to COVID-19 disease remains a pandemic [1]. Disease severity varies significantly from asymptomatic infection to multiple organ dysfunction syndrome (MODS) and death. Critical illness and use of immunosuppressant medications can trigger Herpes Simplex Virus-1 (HSV-1) reactivation, in both immunosuppressed and immunocompetent individuals [1,2]. We describe the case of COVID-19-induced MODS secondary to systemic HSV-1 reactivation. The patient received dexamethasone and sarilumab as part of their COVID-19 treatment. Satisfactory recovery was achieved with antiviral treatment and multi-organ support.

## CASE PRESENTATION

A 49-year-old, immunocompetent and fully vaccinated female patient with past medical history of asthma and obesity (BMI 53.2) presented to the emergency department with dyspnoea at rest and hypoxia following a three-week history of productive cough and progressively worsening dyspnoea. Her symptoms had only partially responded to antibiotics and steroids. Her COVID-19 lateral flow test became positive three days before admission.

**Fig. 1.**
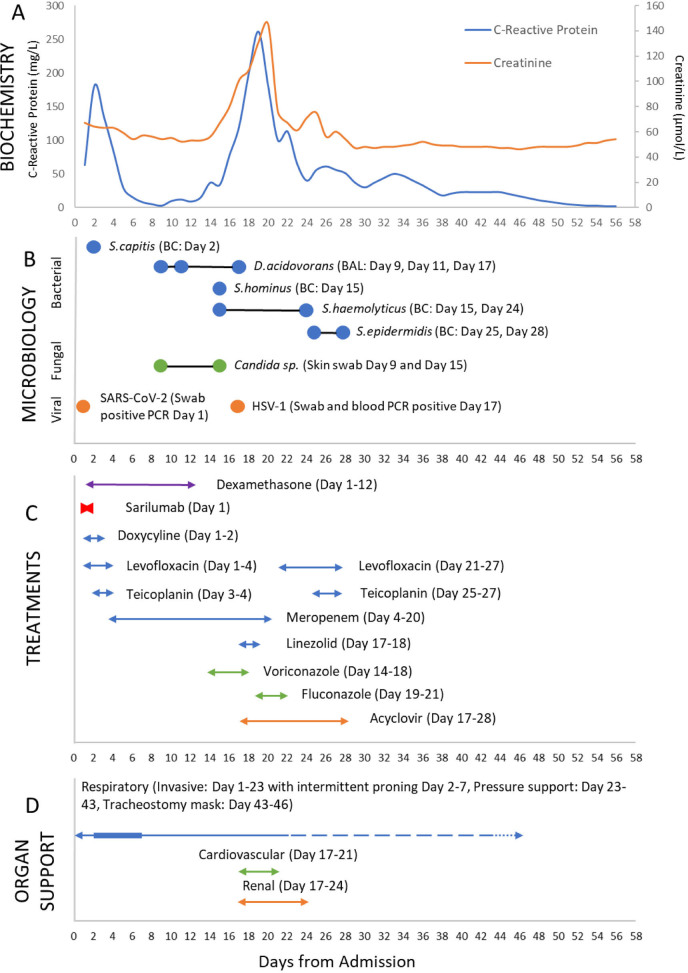
Timeline of events throughout ICU admission. A. Biochemical results (CRP and Creatinine), B. Microbiology results, C. treatments (Blue: bacterial/antibiotic, Green: fungal/antifungal, Orange: viral/antiviral). D. Organ support: controlled mechanical ventilation (continuous line), proning (thick line), pressure support (dash line), and tracheostomy mask (dotted line). Cardiovascular support: noradrenaline infusion. Renal support: CRRT.

On admission, non-invasive ventilation (NIV), nebulised salbutamol and ipratropium bromide, and magnesium sulphate were promptly administered without effect. Due to continuous deterioration the patient was admitted to ICU. The timeline of events, including biochemical and microbiology results, antimicrobial therapies, organ dysfunction, and organ supportive therapies are demonstrated graphically in Figure 1.

On ICU admission, the patient was intubated and ventilated. She was proned daily from day two to seven for 16 hours per day. This was discontinued due to lack of effectiveness. She received 6.6mg IV dexamethasone daily on days one to ten and a dose of sarilumab 400mg IV on day one.

During the first 12 days in ICU, the patient demonstrated gradual improvement in her ventilation and gas exchange (Table 1). Pyrexia resolved and the function of all organ systems except the lungs was normalised. She remained intubated and on invasive mechanical ventilation. Multiple-site cultures were tested during her ICU stay for bacterial, fungal and opportunistic infections. All infections were covered with appropriate antimicrobial therapies.

**Table 1.** Severity scores on admission, ogan support parameters and biochemical test values at key stages throughout ICU admission

	Test	Admission	Day 7	Day 17: HSV-1 Positive	Day 28: Completion of Acyclovir	Day 49: ICU Discharge
**Severity Score**	MODS	8	N/A	N/A	N/A	N/A
	SOFA	8	N/A	N/A	N/A	N/A
	APACHE-II	14	N/A	N/A	N/A	N/A
**Respiratory Support**	FiO2 (%)	60	35	70	35	21
	PEEP (cmH20)	16	16	15	8	N/A
	Pressure Support (cmH20)	N/A	N/A	N/A	15	N/A
	Mode of Ventilation	Volume Control	Volume Control	Volume Control	Pressure Support	No Support
**Cardiovascular Support**	Noradrenaline dose (mcg/kg/hour)	0	0	0.03	0	0
**Renal Support**	Filtration Rate (ml/kg/hr)	0	0	25	0	0
**Laboratory Findings**	Hb (g/L)	142	108	100	90	110
	PLT (×10^9^/L)	209	216	171	231	237
	WCC (×10^9^/L)	8.2	5.4	10.3	10.1	7.5
	Neutrophils (×10^9^/L)	7.3	3.9	7.1	7.4	4.7
	Lymphocytes(×10^9^/L)	0.6	1.3	2	1.7	2.3
	PT (seconds)	12.9	13.4	14.3	15.9	N/A
	APTT (seconds)	28.9	23.6	29.7	31.1	N/A
	INR	1.0	1.1	1.1	1.1	N/A
	Urea (mmol/L)	3.4	11	11.7	5.6	3.3
	Creatinine (μmol/L)	67	57	101	47	48
	eGFR (ml/min/1.73m^2^)	>90	>90	58	>90	>90
	Na^+^ (mmol/L)	136	143	144	141	137
	K^+^ (mmol/L)	4.2	4	4.2	4.1	3.1
	ALT (iu/L)	53	73	44	33	29
	ALP (iu/L)	58	47	64	61	63
	Bilirubin (μmol/L)	9	10	14	10	8
	Albumin (g/L)	39	31	31	30	34
	CRP	63	8	121	37	11
	pH	7.4	7.54	7.41	7.45	N/A
	Lactate (mmol/L)	1.6	1.8	1.7	1.6	N/A
	pO2 (kPa)	9.7	8.7	12.6	8.7	N/A
	pCO2 (kPa)	5.7	6.4	8.3	5.1	N/A

**Fig. 2.**
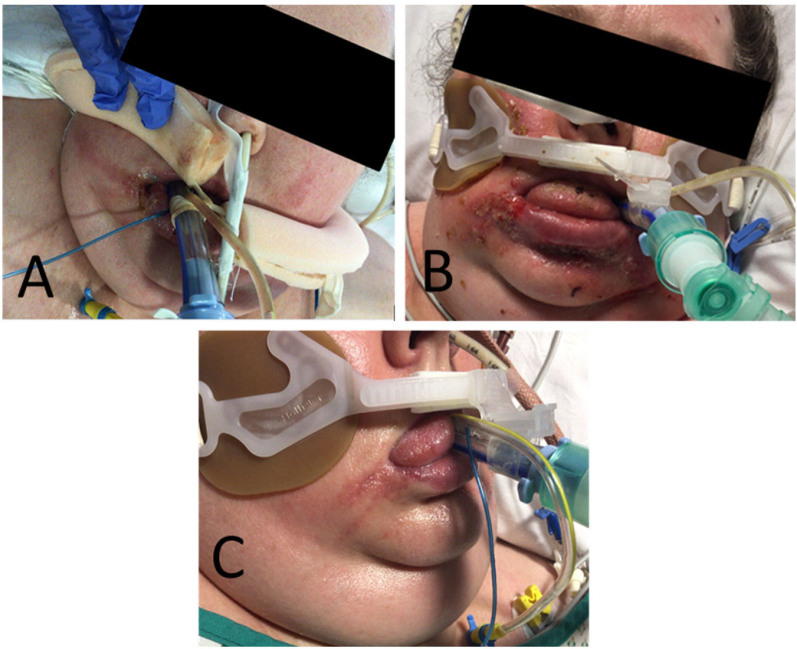
Progression of facial rash A) day 8, B) day 17, and C) Day 30

**Fig. 3.**
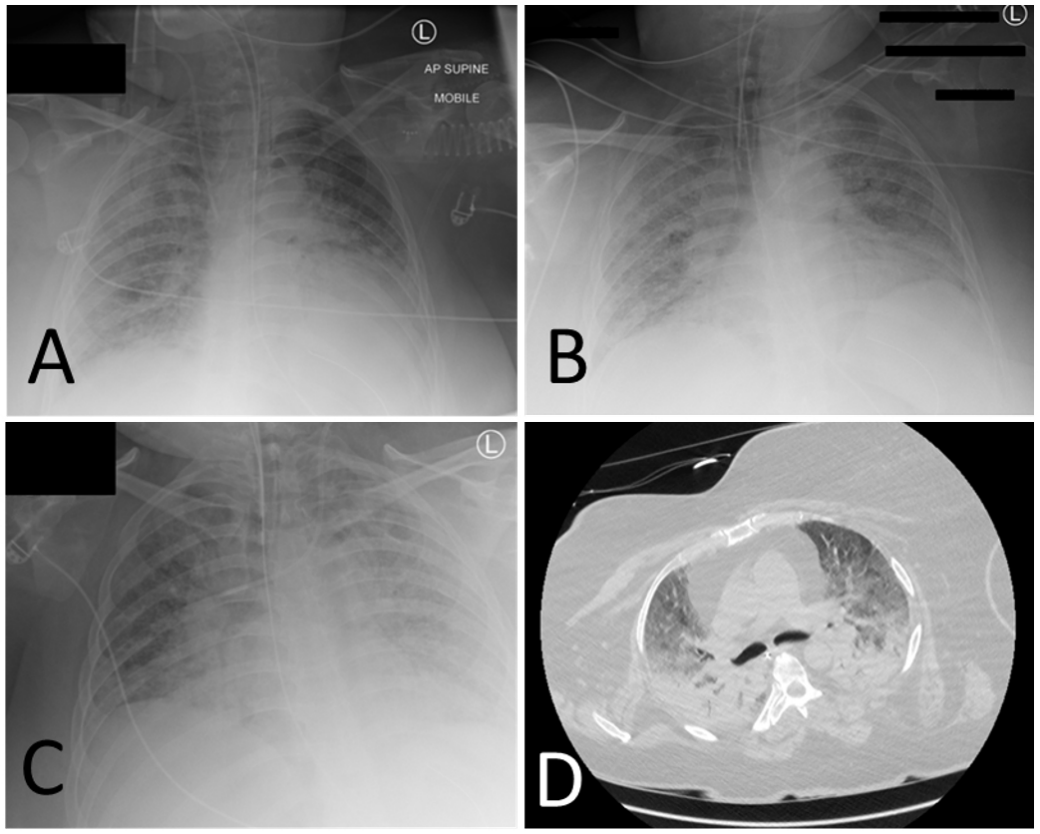
Chest x-rays from A) day 8, B) day 17, and C) Day 30. D) Computed tomography pulmonary angiogram on day 9 demonstrating widespread ground glass appearances consistent with COVID pneumonitis.

From day 13, the patient deteriorated with pyrexia (38.4-39.5°C) and worsening skin lesions over lips, tongue, and cheeks (Figures 2A, 2B). C-reactive protein (CRP) levels began to increase. MODS developed with a further deterioration of respiratory function, cardiovascular compromise requiring a noradrenaline infusion, and acute kidney injury stage two which, due to refractory fluid overload, required continuous renal replacement therapy (CRRT) at 25ml/kg/hour. All medication doses were adjusted according to renal function. Bilateral diffuse infiltrates in chest x-ray worsened while computed tomography showed bilateral dense consolidations and extensive ground glass changes (Figure 3).

Skin lesion swabs and blood polymerase chain reaction (PCR) for HSV-1 were performed which proved positive in high titres in all specimens (cycle threshold 18.80 and 35.69 for facial swab and blood PCRs respectively). As such, IV acyclovir was given for 10 days at 10mg/kg (ideal body weight) every eight hours to treat HSV-1 reactivation.

A significant improvement of MODS was noted (Table 1), the pyrexia resolved, facial rash improved (Figure 2C), CRP reduced, vasopressor support weaned off, CRRT was discontinued. Respiratory function improved, and the patient begun weaning off pressure support ventilation. Surgical tracheostomy was performed after an extubation trial failed (due to critical care-induced neuro-muscular weakness). Patient was successfully decannulated on day 48, discharged to the respiratory ward on day 49, and discharged home on day 63.

## DISCUSSION

This case presents the reactivation of HSV-1 during treatment for COVID-19. The pyrexia and MODS coincided with high HSV-1 titres in skin and blood followed by clinical recovery with acyclovir indicating that HSV-1 was the culprit for the secondary deterioration.

Critical illness-induced HSV-1 reactivation is possible in immunocompetent patients. Previous studies in critically ill immunocompetent patients showed serological evidence of HSV reactivation in 68% of cases. However, those studies failed to show a relationship between viral reactivation and clinical outcomes [3,4,5].

In this patient, the reactivation of HSV-1 was the likely reason for the MODS from day 12 onwards which prolonged the duration of mechanical ventilation and length of ICU stay. Previously published evidence suggested approximately 47% of immunocompetent ICU COVID-19 patients had at least one viral reactivation in blood/BAL PCR samples following the initial week of ICU admission [6]. In addition, this reactivation was associated with prolonged mechanical ventilation and ICU stay in those patients [6]. Furthermore, 60-day mortality and incidence of pneumonia were significantly higher in blood/BAL PCR positive COVID-19 patients [2]. Finally, the median time from hospital admission to HSV reactivation was 19 days and there was an increased likelihood of HSV reactivation with steroid use [7]. Whilst in our case the cumulative dosing of steroids was not excessive, the potential association with the HSV-1 reactivation and illness cannot be excluded.

IL-6 inhibitor contribution to HSV-1 reactivation is debatable. Given that IL-6 promotes T-cell response, macrophage migration and activation, and IgG isotype switching, this hypothesis is valid theoretically [8,9]. However, this has not yet been demonstrated clinically in alternative uses of IL-6 inhibitors, and a recent cohort study found no association between HSV-1 reactivation and tocilizumab use in COVID-19 [7].

In a cohort study of 50 patients, severe COVID-19 was associated with impaired function of monocytes and dysregulation of type 1 interferon (IFN-1) which can lead to a higher viral load and dysregulated TNF-alpha and IL-6 systemic response [10]. This has the downstream effect of greater systemic inflammatory response whilst reducing the ability to induce innate antiviral signalling pathways. Serial immunophenotyping in COVID-19 patients revealed that IFN-1 dysregulation occurred in the later phases of the infection (beyond 11 days) [11] which coincides with the typical timing at HSV-1 reactivation, as seen in the presented case.

Critical illness-induced immunosuppression (CI-IMS) in immunocompetent patients has been increasingly identified. This has been associated with increased risk for viral reactivation and is considered an indirect consequence of the dysregulated immune response in critical illness to the function of antigen presenting cells and the extracellular levels of TNF-alpha. Specifically, after the reduction of intensity of the initial inflammatory response, a prolonged phase of dominant counter-inflammatory response follows leading to suppression of the inflammatory response and potentially favouring the development of immuno-paralysis [12,13]. The effect of this persistent (beyond 2 days) anti-inflammatory response to the antigen presenting cells is downregulation of HLA molecules in monocytes, leading to impairment of antigen presentation and signalling, especially when HLA molecular concentrations decrease to <8000 molecules/cell. This can lead to impaired activation and function of natural killer cells, adverse effects in viral cytotoxicity, increased risk of viral reactivation and risk of development of secondary infections, prolongation of complicated critical illness, and increased mortality [13].

Given the timing of the HSV-1 induced inflammatory peak with resulting new onset of MODS, this new deterioration in patient’s condition does not coincide with the expected effect of CI-IMS. However, CI-IMS can theoretically develop at earlier stages after the administration of combined immunosuppressive treatments like dexamethasone and sarilumab, as in this case. Their immunosuppressive effects combined with the prolonged single organ failure in the patient might have contributed to an early viral reactivation and MODS. Further research is needed to elucidate the mechanisms of these complex interactions between the effects of a virus on the immune system, the immune system response, and its implications on inflammatory/anti-inflammatory regulation during critical illness.

Regarding treatment for HSV-1 reactivation, in a randomised-controlled-trial investigating ventilated patients with oropharyngeal HSV-1 reactivation, acyclovir did not decrease number of ventilator-free days, but did show a trend towards lower 60-day mortality [14]. However, a meta-analysis showed improvement in 30-day mortality for ventilated ICU patients and a trend towards reduced ICU mortality [15].

In critically unwell immunocompetent COVID-19 patients there is currently limited evidence on: a. how to identify risk for HSV-1 reactivation, b. what risks factors are associated with the increased likelihood of reactivation, c. how to prevent HSV-1 reactivation and d. if, when, or in which patients is acyclovir is indicated. Further research is warranted to guide the management of these clinical uncertainties.

## CONCLUSION

An immunocompetent 48-year-old patient was admitted to the ICU with acute respiratory distress secondary to COVID-19. Despite an initial period of improvement, the patient developed new onset MODS. Clinical and serological evidence established the diagnosis of HSV-1 reactivation which considered the most probable cause of MODS. Treatment with high-dose acyclovir led to clinical recovery of the patient. This case demonstrates the low threshold to suspect HSV-1 reactivation and systemic illness in immunocompetent critically ill patients with COVID-19 who are treated with dexamethasone and IL-6 inhibitors. Prompt diagnosis and treatment with high-dose acyclovir can significantly decrease the morbidity and mortality associated with this complication.
